# Breast abscess due to *Nannizziopsis obscura* in an immunocompromised renal transplant patient after travel to Nigeria: case report and review

**DOI:** 10.1186/s12879-022-07958-3

**Published:** 2023-01-24

**Authors:** Nicolás Jiménez-García, Fernando Fernández Sánchez, Celeste María Guillén Rodríguez, María del Mar Castilla Castellano, Alfonso Del Arco Jiménez

**Affiliations:** 1 Internal Medicine Department, Hospital Costa del Sol, Marbella, Spain; 2 Microbiology Department, Hospital Costa del Sol, Marbella, Spain; 3 Obstetrics and Gynecology Department, Hospital Costa del Sol, Marbella, Spain; 4 Nephrology Department, Hospital Costa del Sol, Marbella, Spain

**Keywords:** *Nannizziopsis obscura*, *Nannizzia obscura*, Breast fungal abcess, Immunocompromised, Renal transplant, Nigeria

## Abstract

**Background:**

*Nannizziopsis* is a genus of fungi with several known cases in reptiles of pyogranulomatous infections at cutaneous and musculoskeletal level, of rapid and fatal evolution. There are few cases of this genus described in humans, mainly skin affection but also with visceral abcesses, typically in immunosuppressed patients, with a recent visit to Africa.

**Case presentation:**

A 45-year-old woman immunosuppressed after renal transplantation and with a recent visit to Nigeria presented with a painless breast abcess, ulceration to the skin and bleeding, and non hematic telorrhea. The mammogram, also completed with an ultrasound scan, showed a polylobulated nodule, BI-RADS 4C. Due to the suspicion of breast cancer, a core needle biopsy was performed and the pathology study showed abundant presence of fungal spores and hyphae. It was identified by genomic amplification of the internal transcription spacer region-2 and a percentage of similarity with sequences of *Nannizziopsis obscura* from GenBank of 98% was obtained. An empiric treatment with anidulafungin was initiated, and after the surgical resection, it was replaced by isavuconazole, with a total time of treatment of one month.

**Conclusions:**

This is the first case report of a successful treatment of *Nannizziopsis obscura* with isavuconazole, with the shortest time of treatment published for this fungi. We highlighted the importance of referring difficult to diagnose species to reference centers, as well as achieving the most complete resection in order to shorten the antibiotic therapy.

## Background

*Nannizziopsis* is a genus of fungi belonging to the family *Nannizziopsiaceae* of the order *Onygenales* [1], with multiple known and reported cases in reptiles of pyogranulomatous infections at cutaneous and musculoskeletal level, of rapid and fatal evolution. There are few cases of this genus described in humans, mainly skin affection but also visceral abcesses (brain, liver…). Most of the cases come from a French cohort registered between 2004 and 2020 [2]. Mostly, it occurs in immunocompromised groups. Furthermore, the geographical area of greatest risk of acquisition is the African continent, specifically in the western part of Africa. In the cases published in the literature, treatment is mainly done with antifungals belonging to the azole group. *Nannizzia obscura* is a species belonging to this genus and a known cause of infection in humans.

In this paper, we report the first case where isavuconazole is used, and of those cases where the full treatment time is described, the shortest treatment regimen described, with no evidence of recurrence after a follow-up period of six months. Furthermore, this is the first report of a human infection caused by *N obscura* in Spain.

## Case presentation

**Fig. 1:**
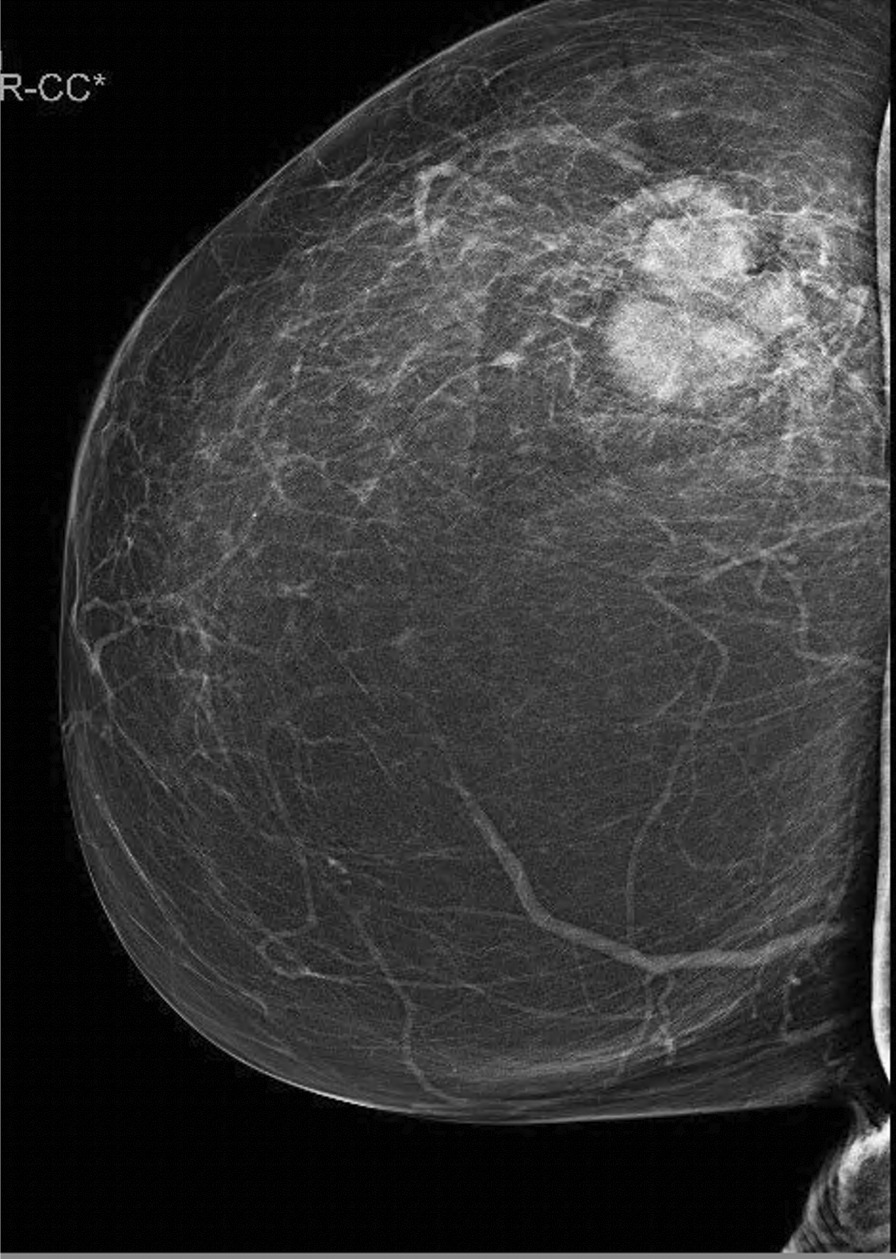
Mammography with evident polylobulated nodular lesion, corresponding to the abscess

A 45-year-old woman consulted in May 2021 in the Emergency Department for a painless, stony breast lump in the right breast of two months of evolution. The tumor had about 4 cm in diameter, was located in the superoexternal breast quadrant and had no associated lymphadenopathies. It had ulceration to the skin and bleeding, some sloughing and grayish pustular exudate. The patient primary care physician requested a mammogram and referral to our breast pathology unit. The mammogram, which was completed with an ultrasound scan, showed a polylobulated nodule, BI-RADS 4C, 40 × 35 mm in diameter, without associated pathological axillary nodes, and ulcerated to the skin (Fig. 1). She consulted the day after the mammography, after starting with non-hematic telorrhea. She did not present fever, chills, nor data of general malaise or systemic involvement.

The patient was a native of Nigeria, with a history of hypertension, dyslipidemia, anemia with sickle cell trait and a history of malaria. She had a myomatous uterus, one full-term pregnancy and five miscarriages. Three years prior to the current episode, the patient had begun follow-up in our center for long-standing chronic kidney disease with no known etiology, and she has been on hemodialysis for 9 months, until receiving a cadaver transplant in May 2019. She develop a Diabetes Mellitus 2 in relation to the use of corticosteroids in immunosuppression scheme, requiring insulinization. The patient's immunosuppressive treatment at the time of this episode consisted of prednisone 5 mg daily, tacrolimus 9 mg at breakfast and 8.5 mg at dinner, mycophenolate 500 mg at breakfast and 250 mg at dinner. During the two years of evolution, she had not presented rejection, and as complications she had presented BK viruria, although without associated BK viremia.

In view of the ulcerated bleeding lesion and with the suspicion of a malign breast disease, a core needle biopsy was performed. The pathology study showed fibrinous and granulation tissue with intense chronic acute inflammatory component, granulomatous reaction, and abundant presence of fungal spores and hyphae, as well as eosinophils and multinucleated giant cells.

**Fig. 2:**
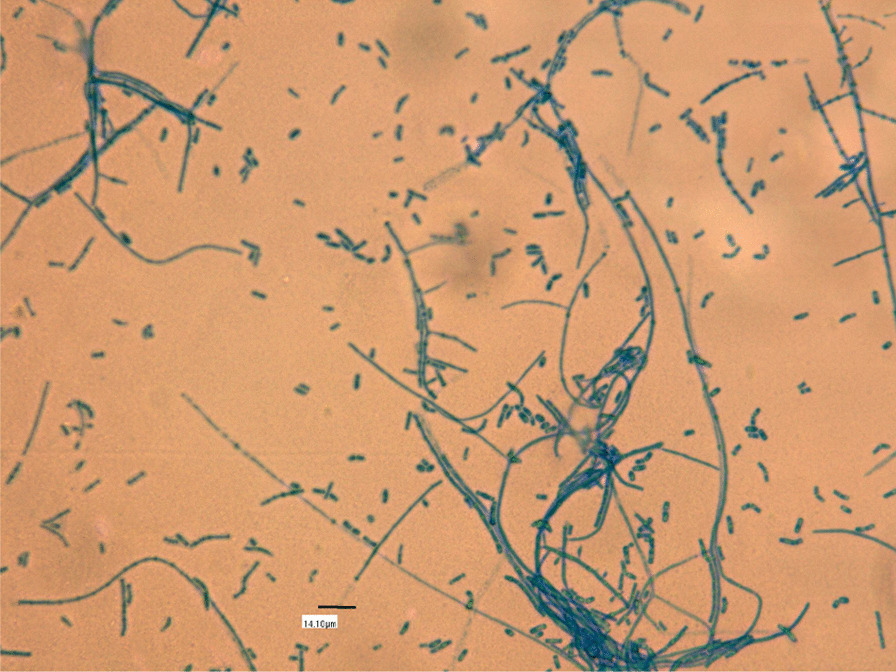
Hyaline septate hyphae with chains of arthro-conidia and sessile microconidia

Given the high suspicion of fungal abscess, a sample of exudate from the lesion was taken and sent to the microbiology laboratory for culture. The sample was cultured in common and specific media for fungal growth (Saboureaud Agar + Chloramphenicol and Potato Glucose Agar—Becton Dickinson®) in aerobic atmosphere at 37 ºC and 30 ºC. Between 48 and 72 h later, growth of white filamentous colonies was observed on the front and back of the plate and, when stained with lactophenol blue, hyaline septate hyphae with chains of arthro-conidia and sessile microconidia were identified (Fig. 2). Preliminary identification was performed with Matrix-Assisted Laser Desorption/Ionization-Time Of Flight (MALDI-TOF)Bruker®and a score lower than 1.25 was obtained, so it was decided to perform molecular identification. It was identified by genomic amplification by polymerase chain reaction (PCR) of the internal transcription spacer region-2 (ITS-2) and, after sequencing, a percentage of similarity with sequences of *Nannizziopsis obscura* from GenBank of 98% was obtained. After that, the sample was sent to the Spanish National Center of Microbiology for microdilution fungigram according to the methodology described in: (a) de Hoog GS, Guarro J, Gené J, Ahmed S, Al-Hatmi AMS, Figueras MJ & Vitale RG (2020) Atlas of Clinical Fungi, 4th edition. Hilversum; and (b) Sutton, Deanna A.; Etc.; Fothergill, Annette W.; Rinaldi, Michael G. Guide to Clinically Significant Fungi), obtaining the MIC results described in Table 1. The sample was registered on GenBank with the code OQ001484.

**Table 1:** Antifungigram

Antifungal	MIC (in mg/L)
Amphotericin B	0.06
Itraconazole	0.25
Voriconazole	0.01
Posaconazole	0.06
Isavuconazole	0.25
Terbinafine	0.5
Caspofungin	0.5
Micafungin	0.03
Anidulafungin	0.03

The patient was scheduled for surgery of the lesion two weeks later. The preoperative blood test showed normal infectious and basic hematological parameters: 5160 leukocytes per microliter (2540 neutrophils per microliter, 1780 lymphocytes per microliter, 40 eosinophils per microliter), 13.1 g per liter of hemoglobin, 285,000 platelets per microliter, with C-reactive protein (CRP) of 8.9 mg/L. In terms of renal function, she had a creatinine of 1.39 mg/dL, equivalent to a Chronic Kidney Disease Epidemiology Collaboration (CKD-EPI) filtration rate of 46 ml/min.

A open biopsy of the lesion was performed without complete excision of the lesion, leaving external drainage through a Penrose catheter. Treatment with anidulafungin was started at that time, still pending definitive typing of the fungus, which at this time is known to be a non-conventional fungus without yeast characteristics, and choosing this option to avoid the possible nephrotoxicity of amphotericin B, which would have been the alternative broad-spectrum antifungal in this case; and to avoid azoles, which would have interacted increasing tacrolimus doses. A total of 14 days of intravenous treatment with anidulafungin 100 mg daily was completed, reducing the tacrolimus dose to reach a target level of 4–5 ng/ml, in agreement with the Nephrology Service. She presented a good evolution during the whole treatment, presenting a residual seroma in ultrasound. During the course of treatment, the fungus was definitively identified as *Nannizziopsis obscura*, with the antifungigram described in Table 1. Given the initial non-complete resection and the immunosuppression status, it was decided to maintain oral treatment for a prolonged period of time, switching to isavuconazole 600 mg for two days and a subsequent dose of 200 mg daily for a total of 14 more days, with good tolerance and acceptance and without side effects.

## Discussion and conclusion

*Nannizziopsis* is a genus of fungi belonging to the family *Nannizziopsiaceae* of the order *Onygenales* [1], thermotolerant keratinophilic ascomycetes, with multiple well known and reported cases in reptiles of pyogranulomatous infections at cutaneous and musculo-skeletal level, of rapid and frequently fatal evolution, in the form of deep, necrotic or granulomatous dermatomycosis, in species such as lizards, geckos, chameleons, iguanas, snakes and crocodiles. Species such as *N guarroi* or *N draconii* are associated with reptile cases, while *N hominis* and *N obscura* seem to be the species associated with human pathology, with more doubt regarding *N infrequens* (with one case described where it seems more of an incidental finding [3]) and *N vriesi* (where there are doubts of identification with N obscura [1]). The sample (biopsy or blood culture) should be processed under standard conditions, and seeded on Saboureaud Agar plus Chloramphenicol and Potato Glucose Agar (Becton Dickinson®) medium in an aerobic atmosphere at both 30 °C and 37 °C for 21 days. White and thinly cottony colonies grow up in the first 48–72 h. Among its characteristics, *N obscura* is a filamentous fungus, with septate smooth-walled hyphae are hyaline, which can present septate conidiophore bearing clavate and sessile conidia, undulate hyphae and barrel-shaped arthroconidia [2]. This may be sufficient for the diagnosis of genus, although we consider essential to complete the study of species by sequencing the colony. Actually, there is a growing intention to use new techniques, such as MALDI-TOF, for the identification of these species [2, 4]. Phylogenetic molecular study of the ITS-2 region to characterize the species should be done(we used a SeqStudio sequencer from Applied Biosystems). The sequence was analyzed in the NCBI database using the blast-n algorithm, identifying the species as *Nannizziopsis obscura* (reference KY771168.1), with 98% homology.

While *N gypseum* has a reservoir in soil, and *N nana* and *N persicolor* have a reservoir in animals, the human pathogenic species have no known reservoir.

There are few cases of this genus described in humans, which are summarized in Table 2. Most of the cases come from a French cohort registered between 2004 and 2020 [2]. Most of the cases described went beyond mere typical dermatophyte conditions, such as ringworm or onychomycosis, probably due to the lack of identification in these types of cases, and the search for the exact species in more complex cases. Mostly, it occurs in immunocompromised groups (HIV positive population, immunocompromised by transplants, hematological malignancies…), especially with involvement of the T lymphoid population. Furthermore, the geographical area of greatest risk of acquisition is the African continent in published cases, specifically in the western part of Africa, in the sub-Saharan region, possibly in semi-arid habitats. Travel times to Africa to the case are from 2 months to 3 years, raising the question of a potential latent form of disease becoming symptomatic with waning immunity.

**Table 2:** Summary of previous cases of Nannizziopsis in literature

Species	Disease	Immunocompromise status	Treatment (and duration, if known)	References	GenBank accesión no. for:
N infrequens	Pneumonia, bronchial wash specimen, localised isolate, not clearly pathogenic	HIV	No	Sigler et al. [5]	AY744467
*N hominis*	Right thigh mass, lung lesion	HIV	Itraconazole	Sigler et al. [5]	KF477215
*N guarroi/hominis*	Inguinal node with disseminated adenopathy initially, after endocarditis, lungs, spleen and kidneys	Immunocompetent	Itraconazole, 2 years	Stchigel et al. [1], reclassified as N hominis in Sigler et al. [5]	HF547876.1
N obscura	Right ankle abcess, osteomyelitis	Immunocompetent	Amphotericin B, 4 months	Stillwell et al. [6]	KF477217
N obscura	Lung infiltration, brain abcess	HIV	Voriconazole	Steininger et al. [7]	HF547869
N obscura	Thoracic collection, lymphadenopathy and skin rash	Renal transplant	Posaconazole, 10 months	Baggott et al. [8]	Non available
N obscura	Brain abcess	Leukemia	No	Nourrisson et al. [9]	KY771168.1
Nannizziopsis sp	Brain abcess	HIV	Amphotericin B 1 month, then voriconazole	Nourrisson et al. [9]	KY771169.1
*Nannizziopsis vriesii/obscura*	Brain abcess	NA	NA	Stchigel et al. [1], reclassified in Sigler et al. [5] as N. obscura	HF547869
*Nannizziopsis obscura*	Liver abcess	HIV	Amphotericin B liposomal	Garcia-Hermoso et al. [2]	MN982937
*Nannizziopsis obscura*	Fungemia, cutaneuous ulcers	Heart transplant	No	Garcia-Hermoso et al. [2]	MN982938
*Nannizziopsis obscura*	Disseminated subcutaneous abcess (legs, back), pulmonary nodules	Diabetes, renal transplant	Posaconazole, 8 months	Garcia-Hermoso et al. [2]	MN982939
*Nannizziopsis obscura*	Suppurated lesions (right ankle) and lung micronodules	Renal transplant	Posaconazole, 2 years	Garcia-Hermoso et al. [2]	MN982940
*Nannizziopsis obscura*	Skin hyperchromic nodules, pulmonary nodules and bronchial ulcerations	Mantle cell lymphoma	Terbinafine, when identifiation voriconazole and liposomal amphotericin B	Garcia-Hermoso et al. [2]	MN982941
*Nannizziopsis obscura*	Mediastinic abcess	Inmunocompetent	Posaconazole, when identification voriconazole	Garcia-Hermoso et al. [2]	MN982942
*Nannizziopsis obscura*	Brain abcess	Renal transplant	Liposomal amphotericin B and fluconazole, then liposomal amphotericin B and voriconazole	Garcia-Hermoso et al. [2]	MN982943
*Nannizziopsis obscura*	Subcutaneous abcess, pulmonary nodules	Renal transplant	Itraconazole, 4 months (ongoing)	Garcia-Hermoso et al. [2]	MN982944
*Nannizziopsis obscura*	Subcutaneous abcess	Renal transplant	Voriconazole, 3 months (ongoing)	Garcia-Hermoso et al. [2]	MT345076
*Nannizziopsis spp.* (not identified)	Disseminated osteomyelitis, pneumonia, brain abcesses	*STAT1* gain of fuction mutation	Amphotericin B and voriconazole, then voriconazole with intention to maintain as prophylaxis	Most et al. [10]	MT104574

There are doubts regarding the route of entry. Although there is frequent cutaneous involvement, skin or nail samples do not recover viable organisms, and the frequency of dissemination described in the literature raises doubts about the possibility of dissemination via inhalation and later through the blood, with the compromise of the skin more like a consequence than as a cause [2].

This fungus belongs to a pathogenic group of difficult typing, with the frequent need to send the sample to a reference center. Many of the published cases are reclassification of previous different identifications [5]. There is cross-reactivity in its typing with species such as *Histoplasma* (*N infrequens*) or *Blastomyces* (*N hominis*) in Accuprobe test set [9], in addition to frequent misidentifications of *Geotrichum* spp., *Trichosporon* spp. or *Tricophyton* spp. by microscopy. When β-D-glucan is performed, this biomarker is elevated, while galactomannan is negative [1]. In cases of disseminated lesions, a positron emission tomography-computed tomography scan (PET-CT) for residual involvement is often performed, helping in the decision to stop treatment [2].

In the cases published in the literature, treatment is mainly done with antifungals belonging to the azole group, including itraconazole, voriconazole and posaconazole, although there are also reports of the use of liposomal amphotericin B. The duration of treatment is not standardized and seems to depend more on the location and clinical form than on the germ itself, but in all cases months of evolution were required. The antifungigrams described in the literature are generally multisensitive to all the treatments described. Our case is the first described in the literature where isavuconazole is used, and of those cases where the full treatment time is described, it is the shortest treatment regimen described, with no evidence of recurrence after a follow-up period of six months.

## Data Availability

All data generated or analyzed during this study are included in this published article [and the additional information files]. The link to the sequence on GenBank is https://www.ncbi.nlm.nih.gov/nuccore/OQ001484.

## Abbreviations

MALDI-TOF: Matrix-Assisted Laser Desorption/Ionization-Time of Flight
PCR: Polymerase chain reaction
ITS-2: Internal transcription spacer region-2
CRP: C-reactive protein
CKD-EPI: Chronic Kidney Disease Epidemiology Collaboration
PET-CT: Positron emission tomography-computed tomography scan
